# Association between subjective health status and frequency of visits to acupuncture clinic: A cross-sectional study

**DOI:** 10.1371/journal.pone.0277686

**Published:** 2022-11-17

**Authors:** Takumi Kayo, Masao Suzuki, Ryuji Kato, Naoto Ishizaki, Tadamichi Mitsuma, Fumihiko Fukuda

**Affiliations:** 1 Department of Acupuncture and Moxibustion, Faculty of Acupuncture and Moxibustion, Meiji University of Integrative Medicine, Nantan, Kyoto, Japan; 2 Department of Kampo Medicine, Aizu Medical Center, Fukushima Medical University School of Medicine, Aizuwakamatsu, Fukushima, Japan; 3 Wallon Acupuncture and Moxibustion Clinic, Sapporo, Hokkaido, Japan; 4 Course of Acupuncture and Moxibustion, Faculty of Health Sciences, Tsukuba University of Technology, Tsukuba, Ibaraki, Japan; University of Huddersfield, UNITED KINGDOM

## Abstract

**Objective:**

There are few studies on the relationship between the frequency of acupuncture use and subjective health status. Therefore, we investigated this relationship using data of a previously performed cross-sectional survey of patients visiting Japanese acupuncture clinics.

**Methods:**

This study used data from a cross-sectional survey conducted in 2011 on patients visiting 180 acupuncture clinics nationwide that were run by members of the alumni association of Meiji University of Integrative Medicine Faculty of Acupuncture and Moxibustion, and did not provide treatment other than acupuncture. We calculated the frequency of visits to acupuncture clinics (< 24 times, 24–47 times, 48–95 times, or ≥ 96 times per year) and the Short Form-36 (SF-36) summary scores (physical, mental, role/social) based on the response of the questionnaire conducted at the time of the survey. Multiple linear regression analysis with multiple imputation was performed with three SF-36 summary scores as the dependent variables, and the frequency of visits to acupuncture clinics as the independent variable.

**Results:**

The questionnaire was distributed to 2,379 outpatients of acupuncture clinics, 1,409 of whom met the criteria and were included in the analysis. More frequent visits to acupuncture clinics were associated with lower scores on all three SF-36 summary scores. Compared to those who visited < 24 times a year, those who visited ≥ 96 times a year had unstandardized regression coefficients (95% confidence interval) of -5.6 (-7.8 to -3.3) for the physical, -2.0 (-3.9 to -0.1) for the mental, and -2.9 (-5.4 to -0.4) for the role/social SF-36.

**Conclusions:**

Frequent visits to acupuncture clinics were associated with poor subjective health status, especially physical health.

## Introduction

With the increasing use of complementary and alternative medicine (CAM) worldwide, studies on the characteristics of CAM users have been conducted, and the relationships between subjective health indicators and CAM use have been investigated. For instance, it has been reported that CAM users have worse self-rated health and the Short Form-36 (SF-36) score than non-users [1, 2]. Since acupuncture is a typical modality of CAM that is widely used in Asia, Europe and the United States, research into the characteristics of acupuncture users has been actively conducted [3, 4]. In addition, the relationship between the use of acupuncture and subjective health status has been investigated mainly in the United States, and it has been reported that acupuncture users were two to four times as likely to report poor self-rated health than non-users [5, 6]. However, these reports compared subjective health status between acupuncture users and non-users, and the relationship between frequency of use and subjective health status has not been examined.

There is a survey using data from the 2011 Korean Ministry of Health and Welfare’s Report (KMOH’s Report) on the Usage and Consumption of Korean Medicine in South Korea. It reported that the percentage of Korean medicine users who answered that their subjective health level was "bad" or "very bad" was greater in frequent users who used ≥ 11 times in a period of 3 months compared to non-frequent users (≤ 3 times) [7]. However, in Korea, China, and Taiwan, acupuncture is a part of traditional medicine, and the survey focused on the use of traditional medicine in general (including herbal medicine), not specifically acupuncture use. There have been no studies from these three countries investigating the association between frequency of acupuncture alone and subjective health status. The only study reported was from the United States, and it found no clear association between the frequency of acupuncture use (with a cutoff of six times a year) and self-rated health status [8]. However, it is not appropriate to apply the results of this study to East Asian countries since acupuncture is likely to be used much more often than this cutoff value [9, 10]. In addition, we have not seen any studies evaluating the association between the frequency of acupuncture use and subjective health status other than self-rated health.

Therefore, by utilizing the cross-sectional survey data conducted in 2011 for patients nationwide who visited acupuncture clinics run by members of the alumni association of Meiji University of Integrative Medicine Faculty of Acupuncture and Moxibustion in Japan, we investigated the relationship between the frequency of acupuncture use and the SF-36 score as indicators of subjective health status.

## Methods

### Data source and participants

**Fig 1:**
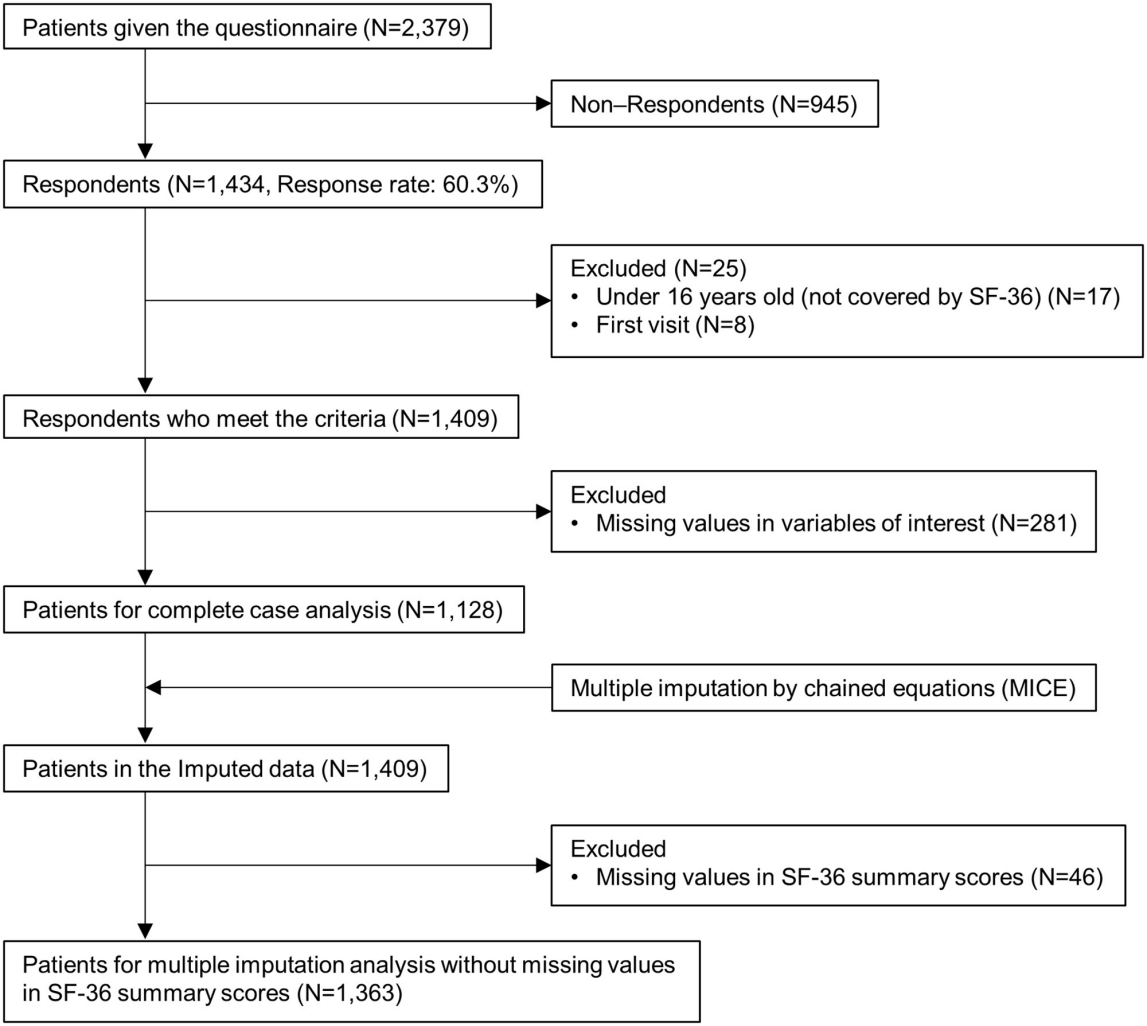
Study flow diagram.

This study used data from a cross-sectional study conducted in 2011 on patients visiting acupuncture clinics [11]. In this cross-sectional survey, acupuncture clinics run by members of the alumni association of the Meiji University of Integrative Medicine Faculty of Acupuncture and Moxibustion were identified from the alumni association membership list as of November 2010, and there were 469 acupuncture clinics which did not administer treatments other than acupuncture (e.g., Judo therapy, massage, or chiropractic). These acupuncture clinics were geographically divided into eight blocks (East Kinki, West Kinki, Kanto, Tokai, Chugoku-Shikoku, Hokushinetsu, Kyushu-Okinawa, and Hokkaido-Tohoku) according to their location. By telephone, we contacted acupuncture clinics which were randomly selected in each block, explained about the study and asked if the clinic had 10 or more patients in the previous 2 weeks. Consequently, 180 clinics, which met the criterion and agreed to participate, were selected as the target acupuncture clinics. Questionnaires on health status, acupuncture treatment status, and basic information were sent to the target acupuncture clinics, whose staff distributed them to visiting patients. The patients brought the questionnaires home, which were then filled in a voluntary and anonymous manner and were directory returned to the investigator. If a patient was a minor, consent was obtained from a surrogate, such as a parent or guardian. The questionnaires were distributed to 2,379 patients from January 24 to February 5, 2011, and collected until February 28, 2011. Valid responses were obtained from 1,434 (60.3%) patients. This study included 1,409 patients, excluding those who visited the clinic for the first time and those who were under 16 years of age (outside the target age of SF-36) (Fig 1).

The study was conducted according to the guidelines of the Declaration of Helsinki, and approved by the Research Ethics Committee of Meiji University of Integrative Medicine (Approval number 2021–013). Patient consent was waived because it had previously been obtained in the initial study [11] whose completely anonymized data was used in the present study.

### Study variables

For the evaluation of clinic visit frequency, the questionnaire asked to choose one from the following options and fill in the brackets:

1. This is the first visit.
2. () times over a period of () week(s).
3. () times over a period of () month(s).
4. () times over a period of () year(s).

With these answers, annual visit frequency was calculated using the following formulae:

1. Patients who chose option 1 were given zero (They were excluded from the study).
2. For option 2, the number of times was divided by the number of weeks and then multiplied by 52.
3. For option 3, the number of times was divided by the number of months and then multiplied by 12.
4. For option 4, the number of times was divided by the number of years.

The annual visit frequency was then separated into four categories; “<24”, “24–47”, “48–95”, and “≥ 96”. These cut-off values were selected based on reports that in Japan twice a month (equivalent to 24 times a year) is the most common frequency of visits, followed by four times a month (equivalent to 48 times a year) [9, 10]. In addition, we set the maximum frequency of visits to ≥ 96 times per year for the following reason: acupuncture treatment programs in Japan are often set as the number of treatments per week or two weeks, and if treatment of once a week (equivalent to about four or five times a month) is not enough, treatment programs are often set up twice a week. Twice a week is equivalent to 8–10 treatments a month, which translates to 96–120 treatments per year.

For the evaluation of subjective health status, the summary scores of the Japanese version of SF-36v2, which is a comprehensive scale, were used [12]. In the original version of SF-36, two summary scores, physical component summary (PCS) and mental component summary (MCS), are calculated. However, the structural concepts of SF-36 in Asian countries such as Japan and Taiwan are different from those in Europe and the US [13], and it has been reported that, in Japan, a structure consisting of the two summary scores of the original version plus the role/social component summary (RCS) score is suitable [14]. In the present study, these three summary scores were used as indicators of subjective health status.

For covariates, variables that may be associated with acupuncture use or subjective health status were selected based on the previous studies [6–8, 15–17]. Covariates included were: age (16–39, 40–59, ≥60), sex (male/female), location of acupuncture clinic (urban/rural), working status (working/not working), annual household income (< 3 million yen, 3–4.9 million yen, 5–6.9 million yen, 7–9.9 million yen, ≥ 10 million yen), education level (lower than high school, high school graduate, higher than high school graduate), number of symptoms (continuous variables), pain (yes/no), treatment costs (0–1,000 yen, 1,001–2,000 yen, 2,001–3,000 yen, 3,001–4,000 yen, 4,001–5,000 yen, ≥ 5,001 yen), duration of visits in years (continuous variables), use of conventional medicine (yes/no), use of other CAMs (yes/no), trust in practitioners (not trust, trust, trust very much), effectiveness of treatment (not effective, effective, very effective). The locations of the acupuncture clinics were considered urban if its address was in the designated city limits as of January 2011, and rural in other cases. The number of symptoms indicated for how many symptoms the patient was receiving acupuncture, and the pain indicated whether the pain was included in the symptoms. The use of conventional medicine indicated whether the patient also visited a clinical department (including Japanese herbal medicine, so-called Kampo) that provides medical care under Japanese public medical insurance coverage for the same symptoms. The use of other CAMs indicated that the patient also used treatments other than “conventional medicine”.

### Statistical analysis

The proportion of missing data for each target variable was calculated. In addition, medians with interquartile ranges for continuous variables, and counts and percentages for categorical variables were calculated. They were also calculated for each visit frequency category, and tested with the Kruskal-Wallis test or chi-squared test.

Missing values for all covariates, including outcome and exposure variables, were imputed under the assumption of missing at random (MAR). One hundred datasets were created by multiple imputation by chained equations (MICE) with predictive mean matching (K = 10) for continuous variables, logistic regression for binary variables, and ordered logistic regression for ordinal variables. After the multiple imputation was performed, the data with missing outcome were excluded [18]. All the subsequent analysis results were aggregated using Rubin’s rule [19]. Before the multiple imputation, 0.5 was added to the number of symptoms in order to eliminate 0 values and logarithmical transformation of the duration of visits and the number of symptoms was performed due to skewed distributions [20].

The relationship between frequency of use and subjective health status was evaluated by linear regression analysis with PCS, MCS, and RCS as the dependent variables and the frequency of visits as the independent variable. After performing crude analysis, multivariate analysis was performed by adding covariates. As a sensitivity analysis, multivariate analysis of the patients with the complete responses with no missing data (complete cases) was performed. All the analyses were performed on STATA version 16 (Stata Corp. College Station, TX, USA).

## Results

Of the 1,409 patients, there were 1,128 complete cases. After multiple imputation, excluding those with missing outcome, the number of patients in the final analysis was 1,363 (Fig 1). Table 1 shows the percentage of patients with missing data for each variable. Among the 1,409 patients, 3.1% did not have data for visit frequency, 3.3% for the SF-36 summary score, and 6.2% for household income, which was the highest.

**Table 1:** Proportion of patients with missing data for each variable.

Variables		Patients with data	Patients with missing data
**Frequency of visits (per year)**	<24	272 (19.3)	43 (3.1)
	24–47	353 (25.1)	
	48–95	539 (38.3)	
	≥96	202 (14.3)	
**Age (years)**	16–39	228 (16.2)	26 (1.8)
	40–59	496 (35.2)	
	≥60	659 (46.8)	
**Sex**	Male	395 (28.0)	18 (1.3)
	Female	996 (70.7)	
**Working status**	Not working	698 (49.5)	54 (3.8)
	Working	657 (46.6)	
**Location of acupuncture clinic**	Rural	1040 (73.8)	0 (0)
	Urban	369 (26.2)	
**Household income (yen)**	<3 million	429 (30.4)	88 (6.2)
	3–4.9 million	384 (27.3)	
	5–6.9 million	222 (15.8)	
	7–9.9 million	176 (12.5)	
	≥10 million	110 (7.8)	
**Education level**	Lower than HS	197 (14.0)	54 (3.8)
	HS graduate	498 (35.3)	
	Higher than HS graduate	660 (46.8)	
**Pain**	No	210 (14.9)	27 (1.9)
	Yes	1172 (83.2)	
**Treatment costs (yen)**	0–1,000	214 (15.2)	26 (1.8)
	1,001–2,000	205 (14.5)	
	2,001–3,000	287 (20.4)	
	3,001–4,000	382 (27.1)	
	4,001–5,000	196 (13.9)	
	> 5,001	99 (7.0)	
**Use of conventional medicine**	No	854 (60.6)	26 (1.8)
	Yes	529 (37.5)	
**Use of other CAMs**	No	1253 (88.9)	26 (1.8)
	Yes	130 (9.2)	
**Trust in the practitioners**	Not trust	95 (6.7)	9 (0.6)
	Trust	614 (43.6)	
	Trust very much	691 (49.0)	
**Effectiveness of treatment**	Not effective	221 (15.7)	23 (1.6)
	Effective	768 (54.5)	
	Very effective	397 (28.2)	
**Number of symptoms**		1382 (98.1)	27 (1.9)
**Duration of visits (years)**		1366 (96.9)	43 (3.1)
**SF-36**	PCS	1363 (96.7)	46 (3.3)
	MCS	1363 (96.7)	46 (3.3)
	RCS	1363 (96.7)	46 (3.3)

HS, high school; CAM, complementary and alternative medicine; SF-36, Short-Form 36; PCS, physical component summary; MCS, mental component summary; RCS, role/social component summary.

Data are n (%).

Table 2 shows patient characteristics by visit frequency of acupuncture clinics. Of the total 1,409 patients, the most common frequency of visits per year was 48–95 (38.3%), followed by 24–47 visits (25.1%), < 24 visits (19.3%), and ≥ 96 visits (14.3%). Participants whose frequencies of visits were ≥ 48 per year were more likely to be older, visited rural clinics, had lower treatment costs, used conventional medicine, felt “Not effective”, and had shorter overall duration of visits. For the SF-36 summary scores, lower PCS and RCS scores were associated with more frequent visits.

**Table 2:** Patient characteristics by visit frequency of acupuncture clinics.

Variables		Values	Frequency of visits (per year)				P-value
			<24	24–47	48–95	≥96	
**Total**		1409	272 (19.3)	353 (25.1)	539 (38.3)	202 (14.3)	
**Age (years)**		58 (44, 69)	55 (41, 66)	55 (43, 66)	60 (44, 70)	60 (46, 73)	**<0.001**
	16**–**39	228	48 (21.1)	64 (28.1)	91 (39.9)	25 (11.0)	**0.007**
	40**–**59	496	115 (23.2)	138 (27.8)	168 (33.9)	73 (14.7)	
	≥60	659	107 (16.2)	143 (21.7)	270 (41.0)	101 (15.3)	
**Sex**	Male	395	85 (21.5)	101 (25.6)	136 (34.4)	64 (16.2)	0.205
	Female	996	185 (18.6)	246 (24.7)	396 (39.8)	137 (13.8)	
**Working status**	Not working	698	128 (18.3)	161 (23.1)	276 (39.5)	103 (14.8)	0.319
	Working	657	140 (21.3)	175 (26.6)	243 (37.0)	93 (14.2)	
**Location of acupuncture clinic**	Rural	1040	175 (16.8)	242 (23.3)	414 (39.8)	172 (16.5)	**<0.001**
	Urban	369	97 (26.3)	111 (30.1)	125 (33.9)	30 (8.1)	
**Household income (yen)**	<3 million	429	82 (19.1)	92 (21.4)	160 (37.3)	70 (16.3)	0.386
	3–4.9 million	384	78 (20.3)	108 (28.1)	133 (34.6)	58 (15.1)	
	5–6.9 million	222	42 (18.9)	62 (27.9)	88 (39.6)	27 (12.2)	
	7–9.9 million	176	38 (21.6)	48 (27.3)	70 (39.8)	20 (11.4)	
	≥10 million	110	21 (19.1)	21 (19.1)	51 (46.4)	16 (14.5)	
**Education level**	Lower than HS	197	34 (17.3)	34 (17.3)	83 (42.1)	32 (16.2)	0.176
	HS graduate	498	95 (19.1)	132 (26.5)	195 (39.2)	67 (13.5)	
	Higher than HS graduate	660	138 (20.9)	177 (26.8)	242 (36.7)	94 (14.2)	
**Pain**	No	210	39 (18.6)	57 (27.1)	83 (39.5)	25 (11.9)	0.682
	Yes	1172	226 (19.3)	291 (24.8)	450 (38.4)	173 (14.8)	
**Treatment costs (yen)**	0**–**1,000	214	6 (2.8)	21 (9.8)	94 (43.9)	79 (36.9)	**<0.001**
	1,001**–**2,000	205	21 (10.2)	45 (22.0)	97 (47.3)	37 (18.0)	
	2,001**–**3,000	287	53 (18.5)	78 (27.2)	109 (38.0)	38 (13.2)	
	3,001**–**4,000	382	99 (25.9)	116 (30.4)	129 (33.8)	29 (7.6)	
	4,001**–**5,000	196	57 (29.1)	56 (28.6)	74 (37.8)	8 (4.1)	
	≥ 5,001	99	29 (29.3)	32 (32.3)	31 (31.3)	5 (5.1)	
**Use of conventional medicine**	No	854	186 (21.8)	242 (28.3)	310 (36.3)	100 (11.7)	**<0.001**
	Yes	529	82 (15.5)	110 (20.8)	223 (42.2)	100 (18.9)	
**Use of other CAMs**	No	1253	244 (19.5)	317 (25.3)	486 (38.8)	182 (14.5)	0.950
	Yes	130	24 (18.5)	35 (26.9)	47 (36.2)	18 (13.8)	
**Trust in the practitioners**	Not trust	95	20 (21.1)	16 (16.8)	32 (33.7)	22 (23.2)	0.127
	Trust	614	119 (19.4)	151 (24.6)	242 (39.4)	86 (14.0)	
	Trust very much	691	131 (19.0)	183 (26.5)	265 (38.4)	92 (13.3)	
**Effectiveness of treatment**	Not effective	221	28 (12.7)	55 (24.9)	91 (41.2)	39 (17.6)	**0.029**
	Effective	768	158 (20.6)	185 (24.1)	309 (40.2)	101 (13.2)	
	Very effective	397	81 (20.4)	109 (27.5)	130 (32.7)	60 (15.1)	
**Number of symptoms**		5 (3, 9)	5 (3, 8)	5 (3, 8)	5 (3, 9)	5.5 (3, 10)	0.461
**Duration of visits (years)**		3 (1, 6)	5 (2, 10)	3 (1, 8)	2 (0.7, 5)	1 (0.3, 3)	**<0.001**
**SF-36**	PCS	46.6 (37.5, 53.0)	48.9 (43.2, 53.5)	47.2 (39.8, 53.4)	45.9 (36.1, 53.2)	41.9 (30.6, 51.7)	**<0.001**
	MCS	48.8 (41.4, 55.3)	49.4 (42.4, 55.2)	48.6 (41.3, 54.3)	48.7 (41.3, 55.5)	48.5 (40.8, 54.8)	0.546
	RCS	49.6 (39.4, 56.5)	51.3 (43.3, 57.1)	51.0 (42.1, 57.6)	49.3 (38.5, 56.3)	45.4 (34.5, 54.5)	**<0.001**

HS, High school; CAM, complementary and alternative medicine; SF-36, Short-Form 36; PCS, physical component summary; MCS, mental component summary; RCS, role/social component summary.

For “Age”, the data show both median (25th percentile, 75th percentile) and n (%). For “Number of symptoms”, “Duration of visits”, and “SF-36”, the data show median (25th percentile, 75th percentile), and for other variables, the data show n (%).

Table 3 shows the results of crude analysis and multivariate analysis with multiple imputation, and multivariate analysis with the complete cases, which was conducted as a sensitivity analysis. The multivariate analysis with multiple imputation showed that more frequent visits to acupuncture clinics were associated with lower scores of all three summaries. This was especially noticeable in the PCS. These results were similar to the results of the multivariate analysis of the complete cases, in which however, the PCS and RCS were slightly higher and the MCS was slightly lower.

**Table 3:** Association between frequency of acupuncture clinic visits and subjective health status.

	Frequency of visits (per year)	PCS		MCS		RCS	
		B (95% CI)	P-value	B (95% CI)	P-value	B (95% CI)	P-value
**Crude analysis with MI N = 1,363**	<24	Ref.		Ref.		Ref.	
	24–47	-1.5 (-3.5, 0.5)	0.142	-0.8 (-2.4, 0.8)	0.308	-0.3 (-2.3, 1.7)	0.785
	48–95	**-4.2 (-6.0, -2.3)**	**<0.001**	-1.0 (-2.5, 0.5)	0.185	**-2.1 (-4.0, -0.3)**	**0.024**
	≥96	**-7.9 (-10.3, -5.6)**	**<0.001**	-1.4 (-3.3, 0.4)	0.126	**-4.9 (-7.2, -2.5)**	**<0.001**
Adjusted analysis with MI* N = 1,363	<24	Ref.		Ref.		Ref.	
	24–47	-1.4 (-3.2, 0.3)	0.111	-0.5 (-2.0, 1.0)	0.493	-0.1 (-2.0, 1.9)	0.928
	48–95	**-2.8 (-4.5, -1.1)**	**0.002**	-1.3 (-2.8, 0.1)	0.068	-0.8 (-2.7, 1.1)	0.397
	≥96	**-5.6 (-7.8, -3.3)**	**<0.001**	**-2.0 (-3.9, -0.1)**	**0.037**	**-2.9 (-5.4, -0.4)**	**0.021**
Adjusted analysis with CC* N = 1,128	<24	Ref.		Ref.		Ref.	
	24–47	-1.3 (-3.2, 0.6)	0.185	-0.6 (-2.2, 1.0)	0.452	0.1 (-2.0, 2.1)	0.942
	48–95	**-2.1 (-3.9, -0.2)**	**0.028**	-1.4 (-2.9, 0.2)	0.081	-0.4 (-2.4, 1.6)	0.670
	≥96	**-5.1 (-7.5, -2.6)**	**<0.001**	**-2.6 (-4.7, -0.6)**	**0.011**	-2.2 (-4.8, 0.4)	0.101

PCS, physical component summary; MCS, mental component summary; RCS, role/social component summary; B, unstandardized regression coefficient; CI, confidence interval; MI, multiple imputation; CC, complete case; Ref, reference.

* Adjusted for age, sex, location of acupuncture clinic, working status, household income, education level, number of symptoms, pain, treatment costs, duration of visits, use of conventional medicine and other CAMs, trust in practitioners, effectiveness of treatment.

## Discussion

In the present study, we analyzed the cross-sectional data of patients who visited acupuncture clinics in Japan to investigate the relationship between the frequency of acupuncture clinic visits and their subjective health status. The results of the multivariate analysis with multiple imputation showed that more frequent visits to acupuncture clinics were associated with lower SF-36 summary scores, especially PCS.

### Comparison with previous studies

Regarding the relationship between the frequency of acupuncture use and subjective health status, a previous study by Schwehr et al. reported using the US National Health Interview Survey data published in 2012 [8]. They found that those who answered "fair or poor" as their health status used acupuncture six times or more in a year, which was more frequent than those with "excellent" health status, although without a statistical significance (odds ratio = 1.9, p-value = 0.078). Of note, their method of evaluating the frequency of acupuncture was different from that used in the current study. Schwehr et al. measured the frequency as the number of acupuncture use within the previous year, but we calculated the annual use based on the response of the questionnaire conducted at the time of the survey. Furthermore, we used PCS, MCS, and RCS of SF-36 as indicators of subjective health status instead of self-rated health. In addition to the difference in the evaluation of visit frequency, choice of cutoff values might have influenced the difference between Schwehr et al.’s study and the present study. Since the number of subjects who used acupuncture less than six times a year was very small in our study (44 subjects, 3.3% of the total), we did not use this as our cutoff value. Compared to the subjects who visited acupuncture clinics < 24 times a year, the subjects who visited 24–47 times a year had lower SF-36 score without a statistical significance; however, the SF-36 score showed a significant difference in the subjects who visited 48–95 times or ≥ 96 times a year.

### Mechanism of the association

A previous national survey of Japanese acupuncture users reported that most of the reasons for acupuncture use were physical problems, especially musculoskeletal problems [15]. The survey also investigated the reasons why acupuncture users continue undergoing acupuncture, and the most common reason was symptom relief. This indicates that many Japanese acupuncture users expect acupuncture to improve their health status, which has impaired due to physical problems. Recent pilot randomized clinical trials have reported that frequently provided acupuncture improves physical symptoms more than infrequent acupuncture [21, 22]. In addition, a meta-analysis of chronic nonspecific low back pain, which is the most common reason for receiving acupuncture, reported that the effect of acupuncture was more likely to be obtained on physical rather than mental health status [23]. It is highly possible that acupuncture users are aware of the effects of acupuncture mentioned in the above reports. Therefore, it is considered that the relationship between the frequency of visits to acupuncture clinics and subjective health status observed in this study may reflect the phenomenon that acupuncture users frequently use acupuncture to improve their physical health status.

### Strengths and limitations

The strengths of this study are as follows. The clinics were selected randomly from acupuncture clinics that had seen 10 or more patients in two weeks. Annual visit frequency was divided into four meaningful categories that were based on survey data of acupuncture usage in Japan [7, 8]. Of 1,409 patients, there were 1,128 cases with no missing data. Only 3.1% of visit frequency data (independent variable) and 3.3% of SF-36 summary scores (dependent variables) were missing. SF-36 results were reported using Asian factor structure (i.e., included a third summary score of role/social component).

Our research has several limitations. First, since the distribution of questionnaires to patients (clinic staff was in charge) was not completely random and there were non-responders (response rate 60.3%), there may be a problem for subject representativeness. Our subjects have some differences compared to previously performed random sampling in Japan. Ishizaki et al. reported that people who experienced acupuncture within the last 12 months were more likely to be female, older, poorly educated, in non-major city, and with a condition whose symptoms include pain [15]. In addition, Yano et al. and Yasuno et al. reported that among those who currently visit acupuncture clinics regularly, “twice a month” was the most common visit frequency, followed by “four times a month” [9, 10]. Our subjects were more highly educated and visited clinics a little more frequently, which may have limited the generalization of our results. Second, the visit frequency information relied on self-reports from the patients. This method requires the patient to estimate how often they receive acupuncture on either a weekly, monthly, or yearly basis, and these estimates may differ from actual visit records. It has been reported that the self-reported use of medical resources is similar to the actual use; however, those with good subjective health tend to underreport the use of resources, and those with poor subjective health are slightly biased toward overreporting [24]. For this reason, we may have overestimated the association between visit frequency and subjective health status. Third, the situation in Japan may have changed between 2011, when the data for this study was obtained, and 2022. According to the Comprehensive Survey of Living Conditions in Japan, the number of people with low back pain and joint pain as the most common symptoms increased between 2011 and 2019 [25, 26]. The presence of these symptoms may have reduced subjective health status and promoted acupuncture use. Thus, the relationship between subjective health status and frequency of acupuncture may be stronger now. Finally, as with all observational studies, the effects of unmeasured bias cannot be ruled out. For example, it has been reported that those whose marital status is widow/widower tend to use traditional medicine frequently and are associated with poor subjective health status [7, 27]. Our data did not investigate marital status.

## Conclusion

We found that frequent use of acupuncture and poor subjective health status were related in outpatients of acupuncture clinics in Japan. Many studies have been conducted on the characteristics of acupuncture users; however, studies focusing on the frequency of acupuncture use are limited. The results of the present study may lead to a better understanding of patients who use acupuncture frequently (or infrequently). Our results may provide very useful information not only for acupuncture providers, but also for medical practitioners who treat acupuncture users and for health policy makers. In future studies, prospective longitudinal studies are needed to investigate the association between baseline characteristics and subsequent visits in order to obtain stronger evidence.

## Supporting information

S1 File The dataset containing the variables used in this study. (XLSX)
